# Regioselective chemoenzymatic syntheses of ferulate conjugates as chromogenic substrates for feruloyl esterases

**DOI:** 10.3762/bjoc.17.30

**Published:** 2021-02-01

**Authors:** Olga Gherbovet, Fernando Ferreira, Apolline Clément, Mélanie Ragon, Julien Durand, Sophie Bozonnet, Michael J O'Donohue, Régis Fauré

**Affiliations:** 1 Toulouse Biotechnology Institute, Bio & Chemical Engineering (TBI), Université de Toulouse, CNRS 5504, INRAE 792, INSA de Toulouse, 135 Avenue de Rangueil, 31077 Toulouse, France

**Keywords:** esterase, feruloylated conjugates, hydrolysis, lipase, transesterification

## Abstract

Generally, carbohydrate-active enzymes are studied using chromogenic substrates that provide quick and easy color-based detection of enzyme-mediated hydrolysis. For feruloyl esterases, commercially available chromogenic ferulate derivatives are both costly and limited in terms of their experimental application. In this study, we describe solutions for these two issues, using a chemoenzymatic approach to synthesize different ferulate compounds. The overall synthetic routes towards commercially available 5-bromo-4-chloro-3-indolyl and 4-nitrophenyl 5-*O*-feruloyl-α-ʟ-arabinofuranosides were significantly shortened (from 7 or 8 to 4–6 steps), and the transesterification yields were enhanced (from 46 to 73% and from 47 to 86%, respectively). This was achieved using enzymatic (immobilized Lipozyme^®^ TL IM from *Thermomyces lanuginosus*) transesterification of unprotected vinyl ferulate to the primary hydroxy group of α‐ʟ‐arabinofuranosides. Moreover, a novel feruloylated 4-nitrocatechol-1-yl-substituted butanetriol analog, containing a cleavable hydroxylated linker, was also synthesized in 32% overall yield in 3 steps (convergent synthesis). The latter route combined the regioselective functionalization of 4-nitrocatechol and enzymatic transferuloylation. The use of this strategy to characterize type A feruloyl esterase from *Aspergillus niger* reveals the advantages of this substrate for the characterizations of feruloyl esterases.

## Introduction

The development of “white biotechnology” is underpinned by advances in enzyme discovery and engineering, areas that are being driven by metagenomics and in vitro-directed enzyme evolution. These techniques procure massive discovery or the creation of new enzyme-encoding sequences, filling up databases with a wealth of information. However, while resolving an early step in the discovery pipeline, these techniques progressively create a new bottleneck regarding enzyme characterization. Therefore, there is a pressing need to extend the enzymologist’s toolbox, providing informationally rich high-throughput screens that can not only attribute an activity to putative enzymes but also procure some qualitative details on enzyme properties. In this respect, the availability of easy-to-use chromogenic substrates that can provide both qualitative and quantitative assays and are compatible with automatized protocols is a crucial issue.

Feruloyl esterases (Faes; EC 3.1.1.73 and the family CE1 of the CAZy classification [1]) are of interest, both because of their role in the deconstruction of complex plant-based materials and also as synthetic tools for the preparation of bioactive compounds with potential antioxidant properties [2–5]. Operating via a two-step serine protease mechanism involving a conserved Ser-His-Asp/Glu catalytic triad [6–7], Faes catalyze the hydrolysis of ester bonds linking hydroxycinnamoyl groups to the glycosyl moieties of plant-based polysaccharides, such as arabinoxylans and arabinans. In this respect, Faes are important components of plant-cell-wall-degrading enzymatic arsenals since the hydrolysis of *trans*-ferulate–polysaccharide linkages contributes to the breakdown of intermolecular bonds that structure the lignocellulosic matrix. Moreover, Faes are useful tools to obtain commercially relevant ferulic acid, which represents up to 3 wt % of plant the cell wall dry weight [8].

So far, the detection and characterization of Faes has mainly relied on either the use of HPLC or on UV–visible spectrophotometry, using natural or synthetic compounds [9–10]. The latter, which are used in high-throughput screening (HTS) assays, fall into three categories. The simplest are feruloyl esters of chromogenic moieties [11–14], such as *p*-nitrophenol or short-chain alkyl groups (e.g., methyl ferulate). More elaborate and biologically relevant substrates contain a feruloylated ʟ-arabinofuranosyl moiety [12,15–17]. These structurally more complex compounds are obtained using multistep syntheses, considerably limiting the availability. Moreover, they might be specific to certain subcategories of feruloyl esterases [18–20], and their use involves a challenging tandem reaction [21]. Finally, the synthesis of other, more generic esters that can be used to assay esterases, including Faes, and lipases have been reported [22–23].

In this work, we revisit the preparation of simple feruloylated substrates, such as the 5-bromo-4-chloro-3-indolyl and 4-nitrophenyl 5-*O*-feruloylated α-ʟ-arabinofuranosides **1a** and **1b**. Although these substrates are commercially available, their synthesis involves 7 or 8 steps [15–17]. This contributes to their rather high retail costs (e.g., as of July 29th, 2020, 2500 and 778 € per 100 mg for **1a** and **1b**, respectively), which are approximately 19- and 14-fold higher than non-feruloylated precursors. Therefore, our aim was to simplify the synthesis in order to reduce the cost. Furthermore, we describe the short synthesis of the new feruloylated chromogenic substrate **12**, a molecule that obviates the need for a glycosyl moiety while containing a cleavable hydroxylated linker that mimics the natural geometry and physicochemical properties of glycosidic linkages.

## Results and Discussion

### Chemoenzymatic synthesis of 5-*O*-feruloylated α-ʟ-arabinofuranosides

**Figure 1:**
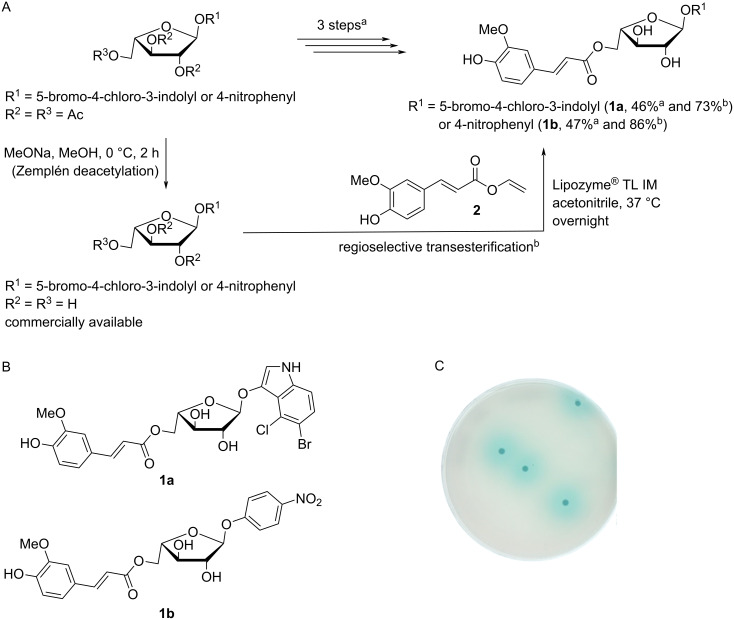
Alternative syntheses (A) and full structures (B) of the 5-bromo-4-chloro-3-indolyl or 4-nitrophenyl 5-*O*-feruloyl-α-ʟ-arabinofuranosidic chromogenic substrates **1a** and **1b** and (C) detection of type A Fae activity on solid agar medium using **1a**. The overall yield of the transesterifications of **1a** and **1b** are reported for both pathways. ^a^3-step pathway. ^b^1-step pathway.

The synthesis of the chromogenic 5-*O*-feruloylated α-ʟ-arabinofuranosides **1a** and **1b** is usually achieved using a multistep pathway that involves trapping the furanose conformation, anomeric activation, glycosidation, regioselective deprotection of the primary hydroxy group, feruloylation, and final deprotection to yield the target molecule [12,15–17]. Additionally, the temporary protection of the functional groups is sometimes used during the synthesis in order to facilitate certain steps. Using an alternative approach, we employed a one-step regioselective transesterification of the unprotected vinyl ferulate **2** (synthesized in 56% in-house yield and in up to 77% previously reported yield [24–25] in one step on a gram scale) using Lipozyme^®^ TL IM (a commercially available immobilized lipase from *T. lanuginosus* that efficiently catalyzes the transesterification of cinnamates) [26–28] and readily available and reasonably cheap 5-bromo-4-chloro-3-indolyl or 4-nitrophenyl α-ʟ-arabinofuranosides. This afforded the corresponding feruloylated derivatives **1a** and **1b** (Figure 1). The yields (73 and 86% for the indolyl and 4-nitrophenyl derivatives, respectively) characterizing the regioselective enzymatic feruloylation of the primary hydroxy group compare favorably with the previously reported overall yield (46 and 47%, respectively, in three steps) [15–17,29], which relate to the selective enzymatic *O*-5-deacetylation and esterification of the primary hydroxy group and a final deprotection of the 2,3-*O*-acetyl groups of the glycoside and the *O*-acetyl group of the ferulate moiety. The fact that lipase-catalyzed transesterification obviates the need for protection and deprotection is a considerable advantage because the final deprotection in the chemical pathway is complicated by the presence of another ester linkage within the molecules [12–15]. In principle, the method described herein is generic, and thus applicable to other chromogenic α-ʟ-arabinofuranosidic compounds, such as 4-nitrocatechol (4NTC), 2-chloro-4-nitrophenyl, and umbelliferyl derivatives.

To demonstrate the suitability of **1a** for qualitative in situ screening of microbial colonies growing on solid agar medium, the compound (300 µg/mL) was incorporated into top agar, along with an α-ʟ-arabinofuranosidase from *Thermobacillus xylanilyticus* (*Tx*Abf). This provided the means to reveal colored microbial colonies expressing the type A Fae from *Aspergillus niger* (*An*FaeA, Figure 1C). Coloration is the result of successive reactions: (i) release of the free 5-bromo-4-chloro-indoxyl-3-ol by an enzyme cascade, wherein *Tx*Abf-catalyzed cleavage of the glycosidic bond was made possible by the prior release by *An*FaeA of the ferulate moiety [21] and (ii) spontaneous oxidation and subsequent dimerization forming the insoluble blue 5-bromo-4-chloro-substituted indigo dye [15]. In an alternative demonstration, **1a** was also used for colony level detection of FAE using yeast cells (*Yarrowia lipolytica* [30]) that actually coexpressed *An*FaeA and *Tx*Abf (data not shown).

### Investigating the enzymatic transferuloylation reaction of non-glycosidic motifs

While lipase-catalyzed transesterifications of cinnamate or phenolic derivatives onto glycosidic structures have been extensively described [25,27–28,31–33], data related to the regioselectivity of transesterifications of hydroxylated alkyl and/or aryl moieties are sparser [26,34–36]. The extent to which Lipozyme^®^ TL IM catalyzes feruloyl transfer reactions involving substituted benzylic alcohols was thus investigated to establish its usefulness for the preparation of both various polyhydroxylated molecules of interest (e.g., antioxidants) [4,26] and of novel chromogenic feruloylated substrates with various physicochemical features for screening applications. Accordingly, we observed that transesterifications only occurred when using primary benzylic alcohols; no phenol acylation was detected with hydroxynitrobenzylic alcohol (Table 1, no side product of **4** and **9** with transfer on an aromatic secondary alcohol; i.e., phenol), 2-chloro-4-nitrophenol, or 4NTC (data not shown). Additionally, the exact position of the benzyl alcohol affected the transfer, with *ortho*-substitutions (R^1^) displaying a hindered electron-withdrawing group. This led to a low target product yield (i.e., **5**, **6**, **8**, and **9**), a lower reactivity, and/or a higher hydrolysis of the vinyl ferulate into ferulic acid.

**Table 1:** Enzymatic transferuloylation of substituted nitrobenzylic alcohols.

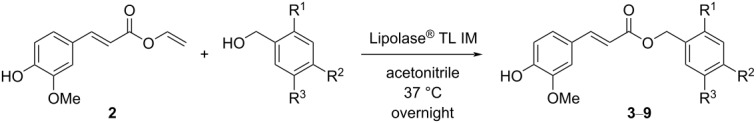					

R^1^	R^2^	R^3^	product	yield (%)^a^	ratio (%)^b^

H	H	NO_2_	**3**	79	10:28:62
OH	H	NO_2_	**4**	76	19:32:49
Cl	H	NO_2_	**5**	44	31:38:31
F	H	NO_2_	**6**	46	33:28:39
H	NO_2_	H	**7**	97	10:24:66
NO_2_	H	H	**8**	–^c^	28:48:24
NO_2_	H	OH	**9**	–^c^	27:64:9

^a^Yield of isolated ferulates after the purification step. ^b^Determined by ^1^H NMR spectroscopy, ratio of the different feruloyl derivatives in the crude reaction mixture: remaining vinyl ferulate, ferulic acid (hydrolysis product), and the ferulates **3**–**9**. ^c^The expected ferulates were confirmed by HRMS analysis, but the low purity of the samples after purification prevented a fine-structural characterization by NMR.

### Synthesis of ʟ-arabinofuranoside-free 4-nitrocatechol-1-yl–linker–ferulate chromogenic substrate **12** and evaluation of **12** as a chromogenic substrate for Fae assays

**Scheme 1:**
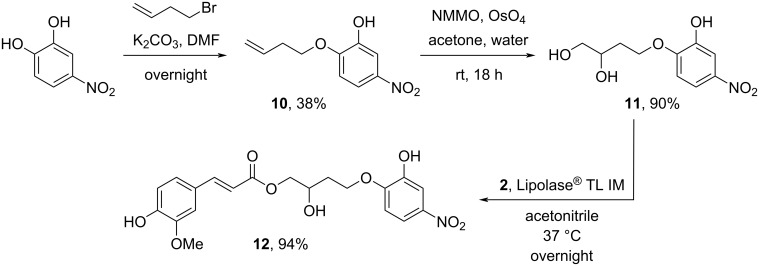
Chemoenzymatic synthesis of (±)-4-*O*-(2-hydroxy-4-nitrophenyl)-1-*O*-*trans*-feruloyl-1,2,4-butanetriol (4NTC–linker–Fe, **12**).

To synthesize chromogenic (*±*)-4-*O*-(2-hydroxy-4-nitrophenyl)-1-*O*-*trans*-feruloyl-1,2,4-butanetriol (4NTC–linker–Fe, **12**), which contains 4NTC bound via a cleavable linker to a ferulate motif, a multistep route was devised (Scheme 1). First, a shorter, more practical pathway towards racemic (*±*)-4-*O*-(2-hydroxy-4-nitrophenyl)-1,2,4-butanetriol (**11**) was developed. Compared to the previously reported 4-step synthesis [37], two drawbacks were circumvented, notably avoiding (i) the preparation of the volatile (*S*)-1-iodo-3,4-*O*-isopropylidene-3,4-butanediol intermediate and (ii) the use of a protected version of the chromogenic linker **11**, either for the extra hydroxy group of the catecholyl moiety or the secondary hydroxy group of the linker [23,37]. It is noteworthy that a racemic mixture of this linker has previously been used to prepare chromogenic substrates for esterases [23,38].

The alkylation of 4NTC with homoallylic bromide under basic conditions gave a mixture of mono- and dialkylated 4NTC derivatives, with **10** (38%) predominating because of the preferential formation of the phenolate at the *para*-position (relative to the nitro group) [39]. Osmium tetroxide-mediated dihydroxylation in the presence of *N*-methylmorpholine *N*-oxide (NMMO) afforded **11** in 90% yield. Finally, the regioselective transferuloylation of the primary hydroxy group of the triol derivative **11** with Lipozyme^®^ TL IM was performed, and the expected chromogenic substrate **12** was isolated in a high yield of 94%. Accordingly, the synthesis of the chromogenic ferulate **12** was achieved in 32% overall yield in 3 steps from commercial reactants (convergent synthesis using a slight excess of the synthesized vinyl ferulate **2**) and without the requirement to perform a final deprotection.

As expected, the investigation of the stability of the chromogenic substrate **12** using UV–visible spectrophotometry revealed that, unlike 4-nitrocatechol-1-yl ferulate (4NTC–Fe), which undergoes spontaneous hydrolysis even at a neutral pH value and 40 °C [13], the presence of the alkyl linker procures a higher stability over a wide pH value range (up to pH 9.0), irrespective of the temperature. This is because in compound **12**, the ferulate moiety is not directly linked to the good leaving group 4NTC (p*K*_a_ = 6.61 [40]). Instead, it is bonded to the linker with a p*K*_a_ value that can be compared either to that of glycerol (p*K*_a_ = 13.61) or ʟ-arabinose (p*K*_a_ = 11.31) [41], meaning that it is a poor leaving group. Moreover, our observations regarding the linker stability are consistent with the known stability of the ester linkages under basic conditions.

**Figure 2:**
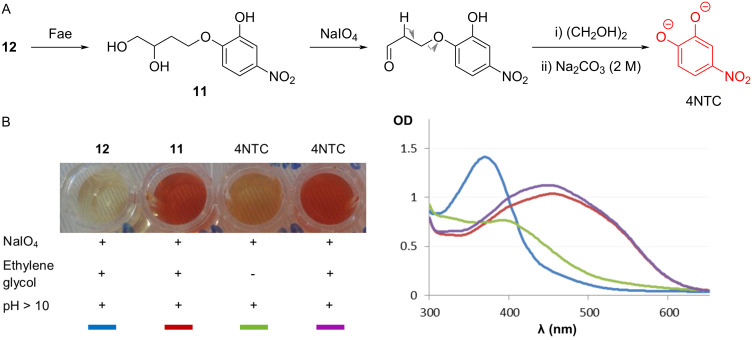
(A) Spectrometric monitoring (at 530 nm) of 4NTC released after the action of Fae on **12** in the presence of sodium periodate and (B) control reactions of the discontinuous assay of the Fae-mediated hydrolysis of **12**. The optical density (OD) was measured during the colorimetric assays. + and − indicate whether the reaction conditions (e.g., pH > 10 or the addition of ethylene) were satisfied in the liquid reaction medium.

The usefulness of 4NTC–linker–Fe (**12**) for the characterization of Faes was investigated (Figure 2), measuring 4NTC release by *An*FaeA [42] at 40 °C. The enzyme-catalyzed reaction leads to the cleavage of the ester bond linking the ferulate to the linker–4NTC moiety, and thus to the accumulation of linker–4NTC. Therefore, working in a discontinuous mode, 4NTC is quantified by submitting samples removed from the reaction to the oxidative action of sodium periodate at 0 °C and registering the absorbance at 530 nm (under alkaline conditions) [23,37–38,43]. Importantly, it is vital to include a stoichiometric amount of ethylene glycol to avoid further oxidation of free 4NTC by sodium periodate (Figure 2B).

The specific activity (SA) of *An*FaeA on 4NTC–linker–Fe (**12**) was determined to be 3 IU/mg of the protein (with IU corresponding to the international unit of the Fae hydrolytic activity), a value comparable to that measured on destarched wheat bran (3 IU/mg) [44] containing 5-*O*-feruloylated α-ʟ-arabinofuranosyl moieties, but lower than that (40 IU/mg, unpublished data) measured using the more labile 4NTC–Fe. Therefore, although 4NTC–Fe is a practical synthetic probe for both the high-throughput screening and the preliminary characterization of the Fae activity [13], 4NTC–linker–Fe (**12**) is almost certainly a better analogue of ferulate linkages found in plant-based structures.

## Conclusion

The use of immobilized Lipozyme^®^ TL IM provides the means to perform the regioselective transesterification of the vinyl ferulate **2** to the primary hydroxy group of benzylic alcohols and polyhydroxylated compounds. Three compounds suitable for the detection and/or characterization of Fae activity were synthesized in a straightforward protocol that holds the potential to greatly reduce the cost of the substrates **1a** and **1b**. Moreover, the enzyme-driven convergent synthesis of **12** affords a novel substrate that is highly suitable for the characterization of feruloyl esterases.

## Experimental

### Materials and general methods

4-Nitrophenyl α-ʟ-arabinofuranosides and 5-bromo-4-chloro-3-indolyl α-ʟ-arabinofuranosides were purchased from Carbosynth and Lipozyme^®^ TL IM (immobilized lipase from *T. lanuginosus*, 250 IUN/g, IUN = interesterification unit) was supplied by Novozymes. The reaction evolution was monitored by analytical thin-layer chromatography using silica gel 60 F_254_ precoated plates (Merck). Spots were visualized using UV light of 254 nm wavelength, followed by soaking in a 0.1% (w/v) orcinol solution containing a sulfuric acid/ethanol/water 3:72.5:22.5, v/v/v mixture, followed by charring. Purification by column chromatography was performed using a Reveleris^®^ flash chromatography automated system (Büchi) equipped with prepacked irregular silica gel 40–63 μm cartridges (FlashPure EcoFlex, Büchi). NMR spectra were recorded on a Bruker Avance II 500 spectrometer at 298 K. Chemical shifts (δ) are given in ppm, with the signals of the residual solvents as the internal references [45]. The coupling constants (*J*) are reported in Hertz (Hz), with singlet (s), doublet (d), triplet (t), doublet of doublets (dd), doublet of doublets of doublets (ddd), broad (br), and quadruplet of triplets (qt) as abbreviations. Analyses and assignments were made using 1D (^1^H, ^13^C, and *J*-modulated spin–echo (*J*_mod_)) and 2D NMR experiments (COSY and HSQC). High-resolution mass spectrometry (HRMS) analyses were performed at PCN-ICMG (Grenoble, France). Optical rotations were measured using a JASCO P-2000 polarimeter at 20 °C.

#### General procedure for enzymatic transesterifications

The enzymatic transesterification steps were performed according to a published protocol [32]. Briefly, Lipozyme^®^ TL IM (1 g, reusable) was added to a solution of alcohol (0.30 mmol, 1 equiv) and the vinyl ferulate **2** (100 mg, 0.45 mmol, 1.5 equiv) in acetonitrile (6 mL). The reaction mixture was stirred overnight at 37 °C, then filtered, the filter cake was washed with acetone, and the filtrate was evaporated to dryness. The residue was recovered in ethyl acetate, and washed three times with saturated aqueous sodium hydrogen carbonate. The combined organic phases were dried over anhydrous sodium sulfate, filtered, and concentrated under reduced pressure. Flash chromatography (gradient of ethyl acetate in petroleum ether from 0 to 50%) afforded the pure ferulates **1a**, **1b**, **3**–**9**, and **12**.

5-Bromo-4-chloro-3-indolyl 5-*O*-*trans*-feruloyl-α-ʟ-arabinofuranoside (**1a**, 107 mg, 0.19 mmol, 73%). Green-yellow foam. The NMR data (CD_3_OD) were consistent with those previously reported [15].

4-Nitrophenyl 5-*O*-*trans*-feruloyl-α-ʟ-arabinofuranoside (**1b**). The application of the general procedure for enzymatic transesterifications was used with commercial 4-nitrophenyl α-ʟ-arabinofuranoside (50 mg, 0.18, mmol, 1 equiv) and the vinyl ferulate **2** (61 mg, 0.28 mmol, 1.5 equiv) to give **1b** (71 mg, 0.16 mmol, 86%) as a yellowish powder. The NMR data (CD_3_OD) were consistent with those previously reported [16–17].

3-Nitrobenzyl *trans*-ferulate (**3**, 78 mg, 0.24 mmol, 79%), white powder. ^1^H NMR (500 MHz, (CD_3_)_2_CO, δ) 8.32 (t, *J* = 1.7, 1H, CH of Bn), 8.21 (ddd, *J* = 8.3, 2.2, 1.1, 1H, CH of Bn), 7.90 (ddd, *J* = 7.6, 1.7, 1.0, 1H, CH of Bn), 7.71 (t, *J* = 7.6, 1H, CH of Bn), 7.68 (d, *J* = 15.8, 1H, CH=CHCO_2_), 7.37 (d, *J* = 2.0, 1H, CH of Fe), 7.17 (dd, *J* = 8.3, 2.0, 1H, CH of Fe), 6.87 (d, *J* = 8.3, 1H, CH of Fe), 6.50 (d, *J* = 15.8, 1H, CH=CHCO_2_), 5.38 (s, 2H, CH_2_ of Bn), 3.92 (s, 3H, OMe); ^13^C NMR (125 MHz, (CD_3_)_2_CO, δ) 167.2 (C=O), 150.3 (C_q_), 149.4 (C_q_), 148.8 (C_q_) 146.7 (CH=CHCO_2_), 140.2 (C_q_), 135.1 (CH of Bn), 130.8 (CH of Bn), 127.3 (2C_q_), 124.2 (CH of Fe), 123.6 (CH of Bn), 123.4 (CH of Bn), 116.1 (CH of Bn), 115.1 (CH=CHCO_2_), 111.4 (CH of Fe), 65.1 (CH_2_ of Bn), 56.4 (OMe); HRESIMS (*m*/*z*): [M − H]^−^ calcd for C_17_H_14_NO_6_, 328.0821; found, 328.0833.

2-Hydroxy-5-nitrobenzyl *trans*-ferulate (**4**, 79 mg, 0.23 mmol, 76%), white powder. ^1^H NMR (500 MHz, (CD_3_)_2_CO, δ) 8.29 (d, *J* = 2.8, 1H, CH of Bn), 8.14 (dd, *J* = 8.9, 2.8, 1H, CH of Bn), 7.68 (d, *J* = 15.8, 1H, CH=CHCO_2_), 7.38 (d, *J* = 1.8, 1H, CH of Fe), 7.17 (dd, *J* = 8.0, 1.8, 1H, CH of Fe), 7.12 (d, *J* = 8.9, 1H, CH of Bn), 6.88 (d, *J* = 8.0, 1H, CH of Fe), 6.52 (d, *J* = 15.8, 1H, CH=CHCO_2_), 5.33 (s, 2H, CH_2_ of Bn), 3.92 (s, 3H, OMe); ^13^C NMR (125 MHz, (CD_3_)_2_CO, δ) 167.5 (C=O), 162.0 (C_q_) 150.3 (C_q_), 148.8 (C_q_), 148.7 (C_q_), 146.6 (CH=CHCO_2_), 141.6 (C_q_), 127.4 (C_q_), 126.3 (2CH of Bn), 124.3 (CH of Fe), 116.5 (CH of Bn), 116.1 (CH of Fe), 115.2 (CH=CHCO_2_), 111.3 (CH of Fe), 61.1 (CH_2_ of Bn), 56.4 (OMe); HRESIMS (*m*/*z*): [M − H]^−^ calcd for C_17_H_14_NO_7_, 344.0770; found, 344.0778.

2-Chloro-5-nitrobenzyl *trans*-ferulate (**5**, 48 mg, 0.13 mmol, 44%), white powder. ^1^H NMR (500 MHz, (CD_3_)_2_CO, δ) 8.42 (br d, *J* = 3.0, 1H, CH of Bn), 8.26 (dd, *J* = 9.0, 3.0, 1H, CH of Bn), 7.81 (d, *J* = 9.0, 1H, CH of Bn), 7.71 (d, *J* = 16.0, 1H, CH=CHCO_2_), 7.40 (d, *J* = 1.8, 1H, CH of Fe), 7.19 (dd, *J* = 8.0, 1.8, 1H, CH of Fe), 6.88 (d, *J* = 8.0, 1H, CH of Fe), 6.56 (d, *J* = 16.0, 1H, CH=CHCO_2_), 5.43 (s, 2H, CH_2_ of Bn), 3.93 (s, 3H, OMe); ^13^C NMR (125 MHz, (CD_3_)_2_CO, δ) 167.1 (C=O), 164.6 (C_q_), 150.4 (C_q_), 148.8 (C_q_), 147.1 (CH=CHCO_2_), 140.6 (C_q_), 137.4 (C_q_), 131.7 (CH of Bn), 127.3 (C_q_), 125.2 (2CH of Bn), 124.4 (CH of Fe), 116.1 (CH of Fe), 114.7 (CH=CHCO_2_), 111.4 (CH of Fe), 63.0 (CH_2_ of Bn), 56.4 (OMe); HRESIMS (*m*/*z*): [M − H]^−^ calcd for C_17_H_13_ClNO_6_, 362.0431; found, 362.0431.

2-Fluoro-5-nitrobenzyl *trans*-ferulate (**6**, 48 mg, 0.14 mmol, 46%), white powder. ^1^H NMR (500 MHz, (CD_3_)_2_CO, δ) 8.43 (dd, *J* = 3.2, 2.9, 1H, CH of Bn), 8.33 (ddd, *J* = 9.0, 4.3, 3.0 Hz, 1H, CH of Bn), 7.67 (d, *J* = 16.0, 1H, CH=CHCO_2_), 7.49 (t, *J* = 9.0, 1H, CH of Bn), 7.36 (d, *J* = 1.8, 1H, CH of Fe), 7.16 (dd, *J* = 8.0, 1.8, 1H, CH of Fe), 6.87 (d, *J* = 8.0, 1H, CH of Fe), 6.49 (d, *J* = 16.0, 1H, CH=CHCO_2_), 5.39 (s, 2H, CH_2_ of Bn), 3.91 (s, 3H, OMe); ^13^C NMR (125 MHz, (CD_3_)_2_CO, δ) 167.1 (C=O), 165.1 (d, *J* = 257.4, C_q_), 150.4 (C_q_), 148.8 (C_q_), 146.9 (CH=CHCO_2_), 127.2 (C_q_), 127.1 (d, *J* = 6.1, CH of Bn), 126.9 (d, *J* = 10.5, CH of Bn), 126.7 (d, *J* = 17.1, C_q_), 124.3 (CH of Fe), 117.7 (d, *J* = 24.3, CH of Bn), 116.1 (CH of Fe), 114.7 (CH=CHCO_2_), 111.4 (CH of Fe), 59.6 (d, *J* = 3.9, CH_2_ of Bn), 56.3 (OMe); HRESIMS (*m*/*z*): [M − H]^−^ calcd for C_17_H_13_FNO_6_, 346.0727; found, 346.0731.

4-Nitrobenzyl *trans*-ferulate (**7**, 96 mg, 0.29 mmol, 97%), white powder. ^1^H NMR (500 MHz, (CD_3_)_2_O, δ) 8.27 (m, 2H, CH of Bn), 7.72 (m, 2H, CH of Bn), 7.69 (d, *J* = 16.0, 1H, CH=CHCO_2_), 7.35 (d, *J* = 2.0, 1H, CH of Fe), 7.17 (dd, *J* = 8.2, 2.0, 1H, CH of Fe), 6.88 (d, *J* = 8.2, 1H, CH of Fe), 6.51 (d, *J* = 16.0, 1H, CH=CHCO_2_), 5.38 (s, 2H, CH_2_ of Bn), 3.92 (s, 3H, OMe); ^13^C NMR (125 MHz, (CD_3_)_2_CO, δ) 167.1 (C=O), 150.3 (C_q_), 148.8 (C_q_), 148.6 (C_q_) 146.7 (CH=CHCO_2_), 145.4 (C_q_), 129.4 (CH of Bn), 127.3 (C_q_), 124.4 (CH of Fe), 124.2 (CH of Bn), 116.0 (CH of Fe), 115.0 (CH=CHCO_2_), 111.4 (CH of Fe), 65.1 (CH_2_ of Bn), 56.4 (OMe); HRESIMS (*m*/*z*): [M − H]^−^ calcd for C_17_H_14_NO_6_, 328.0821; found, 328.0825.

#### Synthesis of the feruloylated 4-nitrocatechol-1-yl-substituted butanetriol 4NTC–linker–Fe (**12**)

2-*O*-(But-3-enyloxy)-5-nitrophenol (**10**). To a solution of 4-nitrocatechol (4NTC, 1.00 g, 6.44 mmol, 1 equiv) in dry DMF (8 mL) were added potassium carbonate (1.06 g, 7.67 mmol, 1.2 equiv) and homoallylic bromide (670 µL, 6.51 mmol, 1 equiv) at 40 °C. After overnight stirring at 40 °C, the reaction mixture was concentrated under reduced pressure. The residue was redissolved in ethyl acetate, washed with a saturated aqueous sodium hydrogen carbonate solution, and brine. The combined organic phases were dried over anhydrous sodium sulfate, filtered, and concentrated under reduced pressure. Flash chromatography (gradient of ethyl acetate in petroleum ether from 0 to 50%) afforded **10** (516 mg, 2.47 mmol, 38%) as a solid. ^1^H NMR (500 MHz, CDCl_3_, δ) 7.82 (dd, *J* = 9.0, 3.0, 1H, CH of 4NTC), 7.80 (d, *J* = 3.0, 1H, CH of 4NTC), 6.90 (d, *J* = 9.0, 1H, CH of 4NTC), 5.91–5.83 (m, 1H, =CH), 5.23–5.16 (m, 2H, CH_2_=), 4.21 (t, *J* = 6.6, 2H, CH_2_O), 2.63 (br qt, *J* = 6.6, 1.6, 2H, CH_2_); ^13^C NMR (500 MHz, CDCl_3_, δ) 151.3 (C_q_), 145.8 (C_q_), 142.1(C_q_), 133.2 (=CH), 118.1 (CH_2_=), 116.8 (CH of 4NTC), 110.4 (CH of 4NTC), 110.1 (CH of 4NTC), 68.5 (CH_2_O), 33.2 (CH_2_); HRESIMS (*m*/*z*): [M − H]^−^ calcd for C_10_H_10_NO_4_, 208.0610; found, 208.0615.

(±)-4-*O*-(2-Hydroxy-4-nitrophenyl)-1,2,4-butanetriol (**11**). A solution of **10** (157 mg, 0.75 mmol, 1 equiv) in acetone/water 2.5–1, v/v (3.5 mL) was treated at 25 °C under stirring with NMMO (106 mg, 0.90 mmol, 1.2 equiv) and osmium tetroxide (2.5 wt % solution in *tert*-butanol, 38 µL) and stirred at room temperature for 18 h. 10% (w/v) aqueous sodium sulfite (0.5 mL) was added, and stirring was continued for 30 min. The product was extracted with ethyl acetate (three times with 10 mL) and washed with brine. The combined organic phases were dried over anhydrous sodium sulfate, filtered, and concentrated under reduced pressure, which afforded the expected compound **11** (165 mg, 0.68 mmol, 90%) as a solid. [α]_D_^20^ 0 (*c* 2.0, CH_3_OH). The NMR data (CD_3_OD) of the racemate **11** were consistent with those previously reported for the pure (*S*)-enantiomer [37] ([α]_D_^20^ −52 (*c* 2.0, CH_3_OH)).

(±)-4-*O*-(2-Hydroxy-4-nitrophenyl)-1-*O*-*trans*-feruloyl-1,2,4-butanetriol (**12**). The application of the general procedure for the enzymatic transesterifications was performed with **11** (70 mg, 0.29, mmol, 1 equiv) and the vinyl ferulate **2** (94 mg, 0.43 mmol, 1.5 equiv) to give **12** (113 mg, 0.27 mmol, 94%) as a white powder. ^1^H NMR (500 MHz, (CD_3_)_2_CO, δ) 7.79 (dd, *J* = 9.0, 2.8, 1H, CH of 4NTC), 7.67 (d, *J* = 2.8, 1H, CH of 4NTC), 7.63 (d, *J* = 16.0, 1H, CH=CHCO_2_), 7.33 (d, *J* = 2.0, 1H, CH of Fe), 7.20 (d, *J* = 9.0, 1H, CH of 4NTC), 7.13 (dd, *J* = 8.2, 2.0, 1H, CH of Fe), 6.87 (1H, d, *J* = 8.2, CH of Fe), 6.40 (d, *J* = 16.0, 1H, CH=CHCO_2_), 4.47–4.42 (m, 1H), 4.40–4.36 (m, 1H), 4.23–4.16 (m, 3H), 3.95 (s, 3H, OMe), 2.18–2.11 (m, 1H), 2.01–1.94 (m, 1H); ^13^C NMR (125 MHz, (CD_3_)_2_CO, δ) 167.4 (C=O), 153.6 (C_q_), 150.2 (C_q_), 148.8 (C_q_), 147.7 (C_q_), 146.0 (CH=CHCO_2_), 142.4 (C_q_), 127.4 (C_q_), 124.0 (CH of Fe), 117.1 (CH of 4NTC), 116.1 (CH of Fe), 115.7 (CH=CHCO_2_), 112.4 (CH of 4NTC), 111.3 (CH of Fe), 110.8 (CH of 4NTC), 69.0 (CH_2_), 67.1 (CH_2_), 66.9 (CH), 56.3 (OMe), 33.7 (CH_2_); HRESIMS (*m*/*z*): [M − H]^−^ calcd for C_20_H_20_NO_9_, 418.1138; found, 418.1139.

#### Screening of Fae(+) microorganisms in solid medium using X–α-ʟ-Ara*f*–Fe (**1a**)

The *Y. lipolytica An*FaeA(+) strain was used to inoculate solid YNB medium (1.7 g/L YNB without casamino acid, 5 g/L ammonium chloride, 20 mL/L oleic acid, 10 g/L ᴅ-glucose, 2 g/L casamino acid, and 15 g/L bacto agar in 100 mM citrate–phosphate buffer at pH 5). The petri dishes were incubated for 48 h at 30 °C and then overlayered with a preparation of 1% (w/v) molten top agar containing the chromogenic substrate **1a** (300 µg/mL and 0.5% DMSO) and *Tx*Abf (2 IU/mL). Once the top agar was solid, incubation at 37 °C for 1 h allowed the color to develop. *Y. lipolytica* strains that contained no *An*FaeA gene were also checked to remain colorless after the addition of the chromogenic substrate and the auxiliary enzyme.

#### Liquid-medium-based colorimetric assays using 4NTC–linker–Fe (**12**)

In a typical experiment, discontinuous enzyme assays were performed in triplicates under buffered conditions (100 mM sodium phosphate at pH 6.0) in the presence of 1.8 mM **12** and 3.6% DMSO, final concentrations. For the assay, this solution was preincubated at 40 °C before *An*FaeA addition. Aliquots (25 µL) were stopped by cooling (at 0 °C) every 6 min over a 24 min period and mixed with 45 µL of a cooled 10 mM NaIO_4_ solution (pH 2.0). After being kept for 5 min at 0 °C throughout, 45 µL of ethylene glycol were added, followed by 135 µL of 2 M Na_2_CO_3_ after 5 min. The OD values at 530 nm were recorded on an Infinite M200 PRO (TECAN) microplate reader. One international unit (IU) of Fae specific activity (SA, expressed in µmol/min/mg or IU/mg) corresponds to the amount of released 4NTC (in µmol) per minute per milligram of protein. Negative controls containing all of the reactants except the enzyme were always included in order to monitor and correct for spontaneous hydrolysis of the substrate. Control reactions containing **12**, **11** and 4NTC without enzyme, and 4NTC without both enzyme and ethylene glycol were also prepared.
